# The Impact of Long-Term Nitrogen Addition on Microbial Community Composition in Three Hawaiian Forest Soils

**DOI:** 10.1100/tsw.2001.450

**Published:** 2001-12-12

**Authors:** Teri C. Balser

**Affiliations:** Department of geological and Environmental Sciences, Stanford University, CA 95706, USA

**Keywords:** lipid analysis, tropical soil, nitrogen deposition, microbial community composition

## Abstract

We evaluated the microbial communities in three Hawaiian forest soils along a natural fertility gradient and compared their distinct responses to long-term nitrogen (N) additions. The sites studied have the same elevation, climate, and dominant vegetation, but vary in age of development, and thus in soil nutrient availability and nutrient limitation to plant growth. Fertilized plots at each site have received 100 kg ha year N addition for at least 8 years. Soil parameters, water content, pH, and ammonium and nitrate availability differed by site, but not between control and N-addition treatments within a site at the time of sampling. Microbial biomass also varied by site, but was not affected by N addition. In contrast, microbial community composition (measured by phospholipid analysis) varied among sites and between control and N-addition plots within a site. These data suggest that microbial community composition responds to N addition even when plant net primary productivity is limited by nutrients other than N. This may have implications for the behavior of forests impacted by atmospheric N deposition that are considered to be “nitrogen saturated,” yet still retain N in the soil.

## INTRODUCTION

During the past several decades, human activity has dramatically increased the quantity of nitrogen (N) added annually to terrestrial ecosystems[1,2]. Because N is often limiting to plant productivity in terrestrial ecosystems, excess N availability can have a large impact on the biodiversity and function of such systems[2]. Most studies of N addition have focused on temperate ecosystems; however, with expected future increases in industrial development and agricultural intensification in the humid tropics, N dynamics in tropical ecosystems will be increasingly important[3,4]. Net primary productivity in tropical ecosystems is often limited by elements other than N, and as a result controls over net primary productivity, litter quality, and competition for N among plants and microbial populations differ from those in temperate zones[4]. For example, N cycling is often more rapid in the humid tropics; mineralized N is converted to nitrate rapidly and is lost readily. In contrast, N added to temperate zone soils tends to be retained in plant and microbial biomass[5].

In addition to a temperate ecosystem focus, most studies of N addition to soils have focused on N pools and fluxes among pools rather than specifically on the microbial populations carrying out processes. The "nitrogen saturation hypothesis" introduced by Aber et al.[6] predicts that ecosystems receiving excess N will initially sequester it, but will eventually reach a saturation point after which the system becomes "leaky"[7,8]; however, results from large-scale, long-term studies of N addition to temperate forests indicate that some of the critical predictions of the N saturation hypothesis are not borne out. It has been suggested that the reason lies partly in our assumptions about the soil microbial community[5]. The fate of N in terrestrial ecosystems will be in large part determined by the microbial community; for example, microbial communities subjected to increased nitrate become dominated by populations capable of readily using nitrate, resulting in increased denitrification[9]. There is an important feedback between N deposition, change in the microbial community, and the fate of N in an ecosystem; however, there has been little microbiology carried out in established, long-term field studies of N deposition (John Aber, personal communication).

In this paper we assess soil microbial community response to long-term N additions in a set of Hawaiian forest soils. We measured microbial community phospholipid fatty acid profiles in soil collected from three experimental forest sites in the Hawaiian archipelago. The sites are at different stages in soil development (300, 20,000, and 4,100,000 years old) and vary naturally in soil nutrient availability (Table 1)[10]. At the youngest and oldest sites plant productivity is limited by N and phosphorus (P), respectively[11,12]. The intermediate-age site has relatively high fertility[13]. The natural fertility gradient provides a range of soils in which to assess the impact of N addition on microbial community composition.

## EXPERIMENTAL METHODS

### Field Sites and Sampling

We used three sites along an age sequence of soils described in detail by Crews et al.[10]. Briefly, the sites are in the Hawaiian archipelago, at approximately 1200-m elevation, with a mean annual temperature of 16°C[10,14]. The sites receive approximately 2500 mm of rainfall annually, which is fairly evenly distributed during the year. The soils at all sites are formed from volcanic tephra and have at least 30 cm of soil above the parent material. The vegetation at each site is native forest dominated by the evergreen tree *Metrosideros polymorpha*[15]. In the upper canopy as well are *Cheirodendron trigynum* and *Ilex anomala*. In the subcanopy are *Coprosma* spp., *Myrsine* spp., *Vaccinium calycinum*, and *Cibotium* spp. tree ferns. Net primary productivity (NPP) at the youngest site has been shown to be limited by N availability. We refer to it from here on as the "N-limited site." At the oldest site, NPP has been shown to be limited by P availability, while at the intermediate-age site soil N and P availability is higher, and NPP is co-limited by N and P. We refer to these sites as "P limited" and "fertile," respectively.

Fertilized plots at the three sites receive 100 kg ha $year^{-1}$ of N (half as ammonium nitrate and half as urea) in two separate additions each year. All three sites (N-limited, fertile, P-limited) have been receiving fertilizer continuously for at least 8 years. The young and old sites have four replicate blocks with 20- × 20-m plots. Plots at the fertile site are centered around individual *Metrosideros* and are 10 m in diameter.

In June 2000 (just prior to the semiannual fertilization), soil samples were collected to 15-cm depth from the control and N-addition treatments at the three sites. This sampling depth included a small amount of mineral soil from each site. Four sets of replicate cores from each plot were composited for a final total of four replicates from each treatment type at each site. At the oldest site it was only possible to obtain three replicates of the N-addition treatment.

### Laboratory Methods

#### *Microbial Fatty Fatty Acid Profiles*

Individual fatty acids are found in the membranes of nearly all microorganisms, but because the relative amount of each fatty acid varies among organism groups, lipid profiles can be used to indicate changes in microbial community composition[16,17]. Furthermore, it has been established that the total amount of lipid extracted from soil is a sensitive index of microbial biomass[18,19,20]. In this paper we present changes in lipid abundance as well as overall profiles in the three fertilized soils.

Phospholipid fatty acids from microbial cell membranes were extracted, purified, and identified using a modified Bligh and Dyer[21] technique. Subsamples of lyophilized soil (5 g each) were extracted in a one-phase extraction mixture containing chloroform:methanol:phosphate buffer (1:2:0.8, v/v/v). We separated phospholipids (polar) from neutral and glycolipids on solid phase extraction columns containing 0.5 g silica gel (Supelco, Inc., Bellefonte, PA). Polar lipids were transesterified by mild alkaline methanolysis. We added 18:0 (octadecanoic methyl ester) and 10:0 (decanoic methyl ester) as internal standards and analyzed samples using a Hewlett-Packard 6890 Gas Chromatograph with an Ultra 2 (5%-phenyl)-methylpolysiloxane column (25 m × 0.2 mm × 0.33 µm) (Hewlett-Packard).

**TABLE 1**
**Site Characteristics**

| Site | Parent Material Age (years) | Treatment | pH | Water Content ($gg^{-1}$) | Ammonium ($\mu gg^{-1}$) | Nitrate ($\mu gg^{-1}$) |
|---|---|---|---|---|---|---|
| N-Limited | 300 | Control | 4.35 ± 0.05* | 1.24 ± 0.16 | 2.27 ± 0.34 | 0.67 ± 0.57* |
| | | N-Addition | 4.08 ± 0.07 | 1.07 ± 0.16 | 2.95 ± 1.18 | 5.83 ± 1.87 |
| Fertile | 20,000 | Control | 3.40 ± 0.16 | 1.40 ± 0.04 | 19.46 ± 8.18 | 128.7 ± 20.0 |
| | | N-Addition | 3.47 ± 0.14 | 1.26 ± 0.05 | 8.78 ± 0.98 | 129.9 ± 21.9 |
| P-Limited | 4,100,000 | Control | 3.18 ± 0.06 | 0.57 ± 0.12 | 2.81 ± 0.39* | 7.36 ± 4.3* |
| | | N-Addition | 3.23 ± 0.23 | 0.75 ± 0.15 | 13.8 ± 1.47 | 30.0 ± 1.64 |

*Note*: Numbers are means ± 1 standard error.
* = Control and N addition are significantly different by t-test ($p < 0.01$).

### Extractable $NH_4^+$ and $NO_3^-$

Soil $NH_4^+$ and $NO_3^-$ were determined by extracting one 20-g, field moist subsample from each of five ecosystem replicates with 100 ml of 2 *M* KCl. Soil suspensions were shaken for 1 h, filtered (Whatman No. 1), and analyzed for $NH_4^+$ and $NO_3^-$ using a flow-injection analyzer (Zellweger-Lachat, Milwaukee, WI). Prior to extraction, filter paper was leached with approximately 100 ml of the extracting solution to remove any $NH_4^+$ or $NO_3^-$ present initially. Nitrate was reduced to nitrite using copperized cadmium and then determined by diazotization. Ammonium was determined by the sodium-salicylate method[22].

### Other Analyses

We determined gravimetric soil water content after 3 days at 105°C. All soil results are reported on an oven-dry basis. Soil pH was determined by electrode in a 1:10 ratio of soil in 0.1 *M* $CaCl_2$.

### Statistical Analyses

Internal standards were used to convert fatty acid peak areas to mole % of total fatty acids extracted. In all subsequent analyses we used only fatty acids that were identifiable and present at 0.5 mole % or higher. We included ten unidentified fatty acids that were present in all treatments. Mole % values sum to a constant value (100%), and thus are not normally distributed. Taking the arcsine of the square root of each fatty acid mole fraction normalizes the data[23]. Transformed values were used for all subsequent analyses. The sum of abundance of all fatty acids is an index of microbial biomass[18,19,20].

We used one way ANOVA followed by Tukey's HSD test, with soil site or "treatment" as the independent variable and microbial community variables as dependent. For all analyses we used JMP in statistical software (SAS Inc.).

## RESULTS AND DISCUSSION

The sites varied in pH, water content, and N availability at the time of sampling (Table 1); however, there were few significant differences by treatment. In the N-limited site, addition of N appeared to result in a decrease in pH and an increase in extractable nitrate. At the P-limited site, N addition resulted in an increase in both extractable nitrate and ammonium. The relatively small differences between control and N-addition treatments may be due to the fact that sampling took place just prior to fertilization. It had been 6 months since N had been last added. We were primarily interested in whether the microbial community had shifted during the course of long-term fertilization; thus, we had purposely avoided "fresh" fertilizer input.

Microbial biomass also varied by site and was not affected significantly by N addition (Fig. 1). The fertile site had the highest biomass, nearly double that of the N- and P-limited sites. Microbial community composition was, however, affected both by site and by treatment (Fig. 2). Ordination of lipid data (principal components analysis) reveals large differences (large separation) among the control plots from each site. The N-addition treatment from each site is quite distant from the respective control treatment (Fig. 2). Adding N to the N-limited site appears to

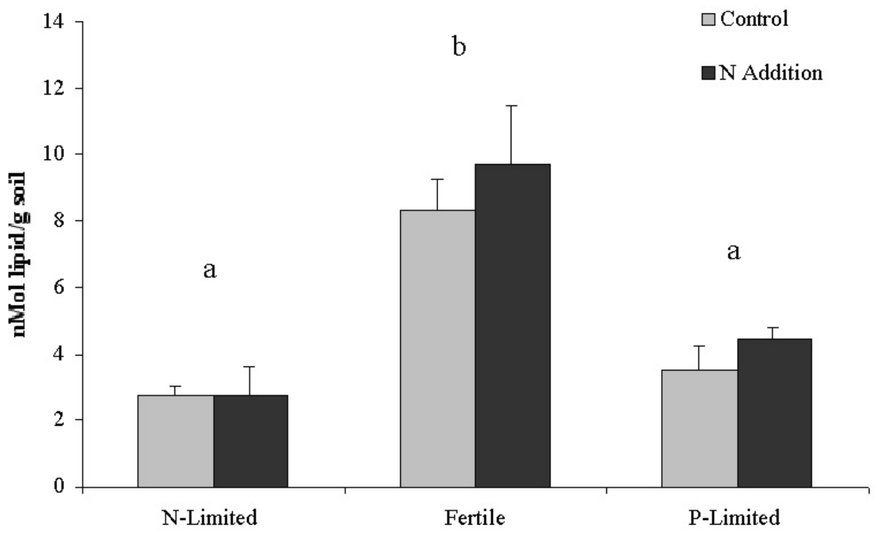

**FIGURE 1.** Total lipid extracted (microbial biomass) at three study sites. Light gray bars are control plots, and dark bars are nitrogen (N)-addition treatment. Error bars are ± 1 standard error of the mean. There were no differences between control and N-addition treatments at any site. Bars from the fertile site were significantly larger than the two other sites ($p < 0.0001$).

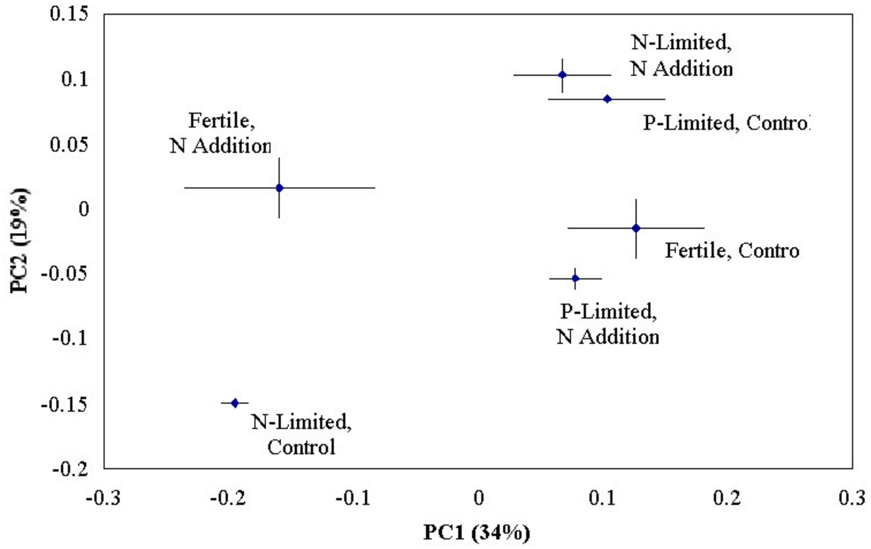

**FIGURE 2.** Ordination of lipid data using principal components analysis. X and Y error bars are ± 1 standard error of the mean. Number in parentheses is the amount of variance accounted for by the principal component.

result in a soil microbial community that is more similar to the P-limited site control soil. Thus it appears that microbial community composition at the youngest site may have been structured by N limitation. When that limitation is relieved, then the community resembles that in the P-limited site. This is consistent with evidence from plant-productivity studies showing that addition of a limiting nutrient to the soil can induce limitation by another element[24].

Addition of N to the P-limited site resulted in a microbial community lipid profile that more closely resembled the fertile site. In this case, it appears that despite the fact that N was not limiting plant productivity at this site, N addition still had a significant effect on the microbial community. Further evidence for this is shown by the response of the microbial community from the fertile site. The community profile from the fertile control plots is not very different than the profile of the P-limited site control plot; however, the profile from the N-addition treatment at the fertile site is very different from any other profile from these sites (Fig. 2). The microbial community thus appears able to respond to N addition even in fertile soils that are not considered N limited. A forest that is N "saturated," having no further vegetation growth response to N availability, may have a microbial community that is still impacted and responding to the N load. Because the microbial community is directly responsible for many aspects of the N cycle, community response may have implications for the long-term response of forest ecosystems to atmospheric N deposition.

## CONCLUSIONS

Microbial communities in three unmanipulated forest soils along a natural fertility gradient differed in biomass and had distinct lipid profiles. While long-term N addition had no effect on microbial biomass at any of the sites studied, it had an impact on microbial community composition. These results suggest that microbial communities in forest soils subject to anthropogenic atmospheric N loading may respond and change even after the system is considered to be "saturated." This study contributes to the growing body of work on microbial communities in tropical soils and to our understanding of how N additions affect ecosystems.

## ACKNOWLEDGMENTS

This research was supported by a grant from the Andrew W. Mellon Foundation. We are grateful to the Division of Forestry and Wildlife and State Parks of the State of Hawaii, Hawaii Volcanoes National Park, and the Joseph Souza Center at Koke'e State Park for access to field sites and logistical support. The author thanks P. Matson, P. Vitousek, H. Farrington, and several undergraduate research assistants for critical feedback and laboratory assistance.

| | | |
|---|---|---|
| Received: | August | 13, 2001 |
| Revised: | November | 14, 2001 |
| Accepted: | November | 14, 2001 |
| Published: | December | 12, 2001 |
